# Are parents of high caries risk Dutch children motivated to brush their children’s teeth? An assessment using the health action process approach questionnaire

**DOI:** 10.1007/s40368-023-00823-0

**Published:** 2023-07-23

**Authors:** K. A. van Nes, C. C. Bonifácio, M. M. van Hunnik, C. van Loveren, I. H. A. Aartman

**Affiliations:** 1 Department of Paediatric Dentistry, Academic Centre for Dentistry Amsterdam (ACTA), Gustav Mahlerlaan 3004- Room 2N41, 1081 LA Amsterdam, The Netherlands; 2 Department of Oral Public Health, Academic Centre for Dentistry Amsterdam (ACTA), Amsterdam, The Netherlands

**Keywords:** Health action process approach, Social cognitive constructs, Caries risk children, Oral health behaviour, Parents

## Abstract

**Purpose:**

To assess the social cognitive constructs of the Health Action Process Approach (HAPA) of parents of high caries risk children to be treated under intravenous sedation (IVS) or with behavioural guidance techniques (BGT), and to assess the changes in these constructs for each treatment group after treatment.

**Design:**

In this cohort study, 160 children aged 3–10 years were allocated by their paediatric dentist to either IVS (77.4%) or BGT. Their parents filled out a HAPA questionnaire, before (T1, *n* = 160), immediately (T2, *n* = 108) and three months (T3, *n* = 71) after their children's dental rehabilitation.

**Results:**

Before treatment, all parents had high scores on all social cognitive constructs. There were no differences in mean HAPA scores between the treatment groups (*p* > 0.05). After treatment, mean scores changed in both groups. In the BGT group, *action self-efficacy* changed from 3.64 (T1) to 3.36(T2) (*p* = 0.027) and to 3.13 (T3) (*p* = 0.021) and *coping self-efficacy* changed from 3.63 (T1) to 3.23 (T2) (*p* = 0.015). In the IVS group, *action planning* changed from 3.25 (T1) to 3.05(T3) (*p* = 0.036) and *action control* changed from 2.58 (T1) to 2.82 (T2) (*p* = 0.012) and to 2.87 (T3) (*p* = 0.006).

**Conclusions:**

High scores on social cognitive constructs of parents of children referred to a paediatric dentist showed that they seem to be motivated to brush their children’s teeth, irrespective of the treatment group. Small changes were observed in the HAPA constructs, however, these are not considered clinically relevant.

**Supplementary Information:**

The online version contains supplementary material available at 10.1007/s40368-023-00823-0.

## Introduction

Dental caries is the most common non-communicable disease in children (Peres et al. 2019). Children may suffer from the consequences of dental caries, such as pain, decreased quality of life and discomfort during dental treatments (Kragt et al. 2016). Since dental caries is the result of frequent consumption of sugar-containing foods, continued presence of dental plaque and lack of topical fluoride, its occurrence and development of caries lesions can be controlled by diet regulation, adequate oral hygiene and use of fluorides (Featherstone 1999). To implement these healthy habits, young children depend on their parents (Pujar and Subbareddy 2013). When it comes to parents of children with dental caries, it is expected that they need support to perform such positive oral health habits (Marshman et al. 2016).

Barriers to achieve healthy oral habits in children are behaviour of the child and lack of parental skills to manage this behaviour (Marshman et al. 2016), doubts and beliefs regarding dental caries and lack of routines (Marshman et al. 2016; Lotto et al. 2020). These factors may hinder the development of healthy oral habits and induce a gap between intention and the actual behaviour. Parents might need guidance in overcoming their barriers to bridge this intention-behaviour gap. A model that may assist parents in changing their oral health behaviour is the Health Action Process Approach (HAPA) (Schwarzer 2008).

**Fig. 1:**
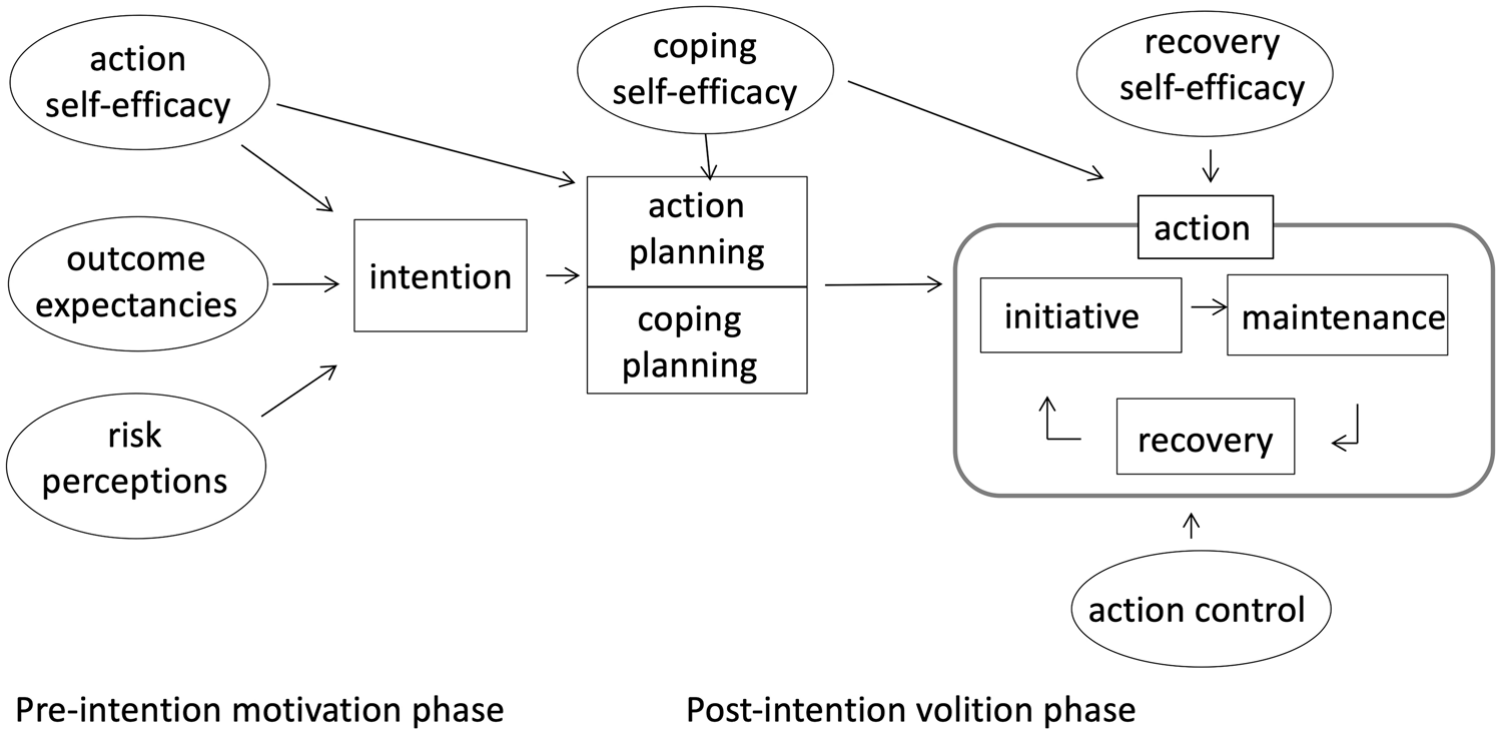
The HAPA model. adapted from Schwarzer (2008)

The HAPA describes two phases to assimilate the new *behaviour*: a motivational phase and a volitional phase (Fig. 1). In the motivational phase the *intention* to perform new healthy behaviour is the result of three social cognitive constructs: *risk perceptions*, *outcome expectancies* and *action self-efficacy*. *Risk perceptions* are parents’ perception of the risks of maintaining the current behaviour to develop a disease. *Outcome expectancies* refer to parents’ understanding of the outcomes of the behaviour. *Action self-efficacy* reflects the confidence parents have in their capacity of starting new healthy behaviour. After the *intention* to change the behaviour is formed, the volitional phase starts. In this phase, three other social cognitive constructs are guiding parents from intention to behaviour: *action planning*, *coping planning* and *coping self-efficacy*. *Action planning* refers to a step-by-step planning to perform the desired behaviour. *Coping planning* covers a parent’s perceived and expected barriers in performing and maintaining the new behaviour coupled with solutions to overcome these barriers. *Coping self-efficacy* refers to parents believe in overcoming the obstacles that arise during the maintenance of the new behaviour. The final phase is related to keeping up the behaviour and consists of *action control and recovery self-efficacy. Action control* is a continuous cycle of adapting new behaviour, perceiving set-backs and recovering from set-backs. It includes a self-regulatory strategy to promote healthy behaviour by monitoring and evaluation of the behaviour. *Recovery self-efficacy* refers to the confidence parents have that they will overcome the set-backs and will restart the new behaviour again (Schwarzer 2008; Schwarzer and Hamilton 2020).

As one of the methods to deliver prevention, HAPA could be a useful tool to guide parents of high caries risk children with difficulties in implementing positive oral health behaviour. A dental health care professional who is aware of the social cognitive constructs of the parent can provide an individual-tailored approach to assist these parents. Therefore, examining baseline HAPA scores of parents of high caries risk children could contribute to successful treatment.

Children with high caries experience can be referred to a paediatric dental practice where they are generally allocated to either treatment under or deep sedation, such as general anaesthesia (GA) and intravenous sedation (IVS) or to multiple chairside sessions using behavioural guidance techniques (BGT) (American Academy of Pediatric Dentistry 2021). Treatment under deep sedation, such as GA or IVS, consists of full dental rehabilitation in one treatment session while the child is deeply sedated (Coté and Wilson 2019). Rehabilitation under deep sedation is mostly performed for children of young age, for children with limited coping skills for dental treatment and/or for children with an extensive treatment plan (Macpherson et al. 2005). The other treatment option, treatment with BGT, is mostly indicated for less extensive treatments and or in children who are expected to cooperate or learn how to cooperate during dental treatment sessions. These children will usually be allocated by the paediatric dentist for treatment with BGT.

Since a high/severe caries experience at a young age is likely to be a result of insufficient oral health behaviour of the parents towards their children (Finlayson et. al. 2019) it might be that the allocation to either treatment with BGT or under deep sedation is related to the motivation and volition of their parents regarding their children’s oral health care. This motivation could be reflected in the HAPA scores of parents before the dental rehabilitation of their children.

The aim of this study is to describe the level of the social cognitive constructs of the HAPA model of parents of high caries risk children referred to a paediatric dental practice and to compare these levels between the parents of children to be treated either under IVS or with BGT. Next, the changes in these constructs after treatment will be assessed for each treatment group.

## Materials and methods

### Ethical statement

The study was approved by the medical ethical committee of the VU University as non-Medical Research Involving Human Subject Act, protocol number 2018-021 and written according to the STROBE guidelines for cohort studies.

### Sample selection

In this prospective cohort study, parents/caregivers (from now on referred to as ‘parents’) of children who were referred to a paediatric dental referral practice in Almere, the Netherlands, for treatment of caries, were invited to participate in this study between May 2018 and April 2020. Dental rehabilitations were carried out under intravenous sedation (IVS) or in multiple chairside treatment sessions with behaviour guidance techniques (BGT) by three paediatric dentists and one paediatric dentist in training. These dentists performed both treatments. Parents were included in the study when their child was healthy, ASA I (American Society of Anesthesiologists 2020), aged 3–10 years, and presented with at least three decayed teeth in the mouth spread among at least three quadrants. The parents should have sufficient understanding of the Dutch language to fill out a HAPA-based questionnaire (online supplementary information S1) and sign the informed consent. Parents were excluded when their child had enamel anomalies other than caries or syndromic anomalies of the teeth. Parents were allowed to participate for one child only. In this referral practice, the allocation ratio for IVS versus BGT was 3:1. The sample size was calculated with G*Power3 (Faul et al. 2007). With a two-tailed independent samples t-test with unbalanced groups (3:1), a 0.4 difference in HAPA mean scores (SD = 0.7) between groups (effect size = 0.57), a significance level of 0.05, a power of 0.80, and an oversampling of 20%, a sample of 159 patients was needed.

### Procedure

**Fig. 2:**
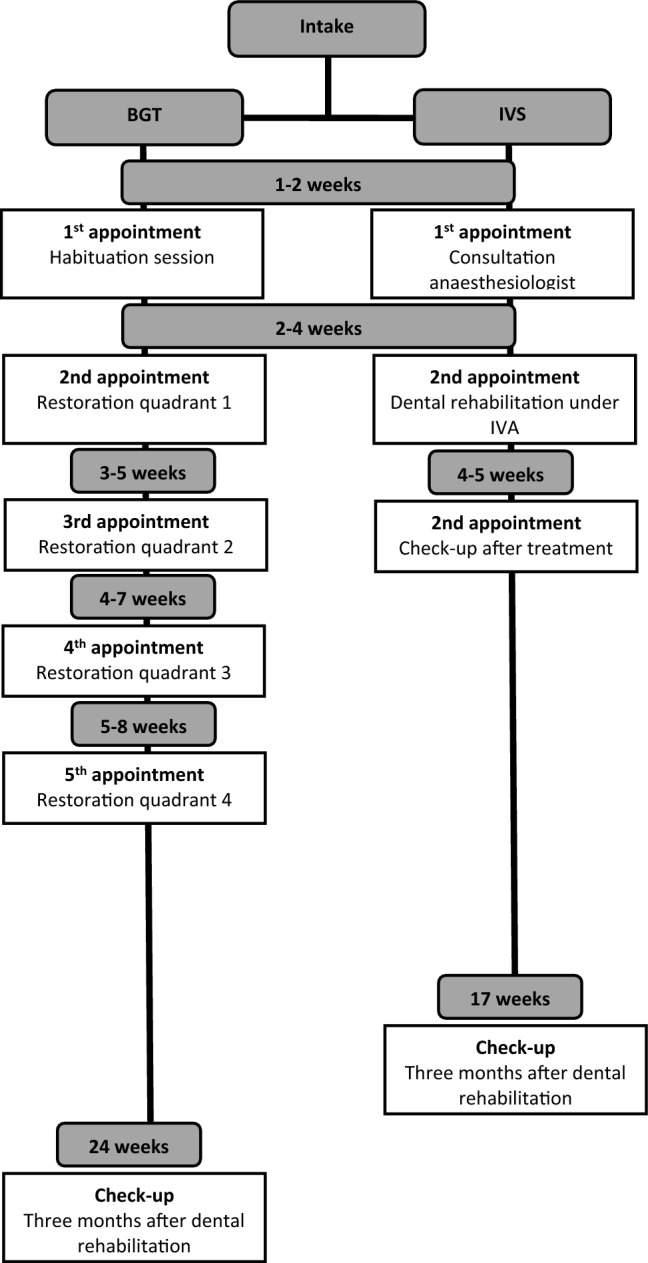
Timeline dental rehabilitation per type of treatment

At intake, parents were informed verbally and in writing by a research assistant about the study procedure. Parents were invited to participate and were assured that they could withdraw from the study at any time without any consequences for the treatment of their child. If parents agreed to participate and signed their informed consent, they were invited to fill out the questionnaire (T1). The paediatric dentist recorded the identification number, date of intake, confirmation of the inclusion criteria, the number decayed (d/D), missing due to caries (m/M), or filled (f/F) teeth in the primary (lowercase) and the permanent (uppercase) dentition together as ‘dmft + DMFT’, and the type of treatment (IVS or BGT) on a registration form. A tooth was considered ‘decayed’ when caries had progressed into the dentine. The paediatric dentist indicated, in agreement with the parents, the type of treatment for the children based on the treatment extension together with the anticipated bearing strength and coping capabilities of the child for the proposed treatment. Hereby, there was no random allocation of the children to one of the types of treatment. The children were treated under intravenous sedation (IVS) or in multiple chairside treatments with behaviour guidance techniques (BGT). The intravenous sedation was performed with propofol and a laryngeal mask. The behavioural guidance techniques that were used include latent inhibition, positive reinforcements, modelling, tell-show-feel-do with shaping and successive approximation and distraction. Children receiving BGT are prepared for treatment during one or more habituation sessions, in which they receive information about the treatment procedure and the dental instruments. The treatments with BGT are split into several sessions to make the treatment acceptable in duration and intensity. Treatments in both groups consisted of restorations (bonded restorations or preformed crowns), pulpotomies, extractions and fluoride applications. Both treatment groups received oral hygiene instructions after dental rehabilitation (T3) as a part of the usual care. Figure 2 presents the timeline of dental rehabilitation per type of treatment. IVS indication was recorded as: young age, extensive treatment plan, limited coping skills, another reason, or a combination of those. Parents were invited to fill out the same questionnaire immediately after dental rehabilitation (T2, i.e., during the last treatment session for BGT and during the check-up after intravenous sedation for IVS) and three months after dental rehabilitation, during their child’s check-up appointment (T3). The follow-up period between T1 and T2 varied depending on the type of treatment and urgency of treatment. The research assistant kept track of the follow-up appointments and prepared the questionnaire for the desk officer to distribute to the parents at the appointments.

### Questionnaire

The questionnaire was a modification of the HAPA-based questionnaire of Gholami and Schwarzer (2014) adjusted from ‘using dental floss’ to ‘brushing the teeth of your children’. It was translated and validated in previous studies (van Nes et al. submitted). The questionnaire consisted of sociodemographic items (only asked for at T1), four items about the parental brushing behaviour of the child and 32 HAPA-based items to assess the eight HAPA constructs*.* The 32 HAPA items could be answered on a Likert scale ranging from *absolutely not true* (1), *not true* (2), *true* (3) to *absolutely true* (4) for the HAPA items of the constructs *intention*, *action self-efficacy*, *coping self-efficacy*, *action planning*, *coping planning* and *action control.* For the constructs *outcome expectancies* and *risk perceptions* the Likert scale ranged from *most unlikely* (1), *unlikely* (2), *likely* (3) to *most likely* (4). Firstly, a mean score of the respective items was calculated for each construct per respondent. As one construct is composed of several items, the mean was calculated by summing the scores on the items of the construct and dividing them by the number of items of that construct, allowing a maximum of one missing value per construct per respondent. For example, for action planning the mean score was calculated by adding the scores of its five items (questions numbered 31, 32, 33, 34, 35) and dividing this score by 5. Then, we calculated the mean score of each construct per type of treatment group. A mean score could range from 1 to 4. When two or more items were missing the respective construct was not included in the statistical analysis.

Sociodemographic items were gender and age of the child and of the mother, country of birth of the child and of both parents, relationship to the child, highest completed education of the mother, and marital status. The level of highest completed education of the mother was dichotomised into ‘low level of education’, including no education, elementary school, lower-level secondary school or other lower levels of education and ‘high level of education’ including higher level secondary school, higher level further education or university. Also, the marital status was dichotomised into ‘single’, including single, divorced or widowed, and ‘with partner’, including married and living together with a partner. The country of birth of the child and of both parents was transformed into the ethnical background of the child.

Several additional items were included. Items regarding parental brushing behaviour of the child consisted of the following questions: who brushes the child’s teeth (‘child only’ or ‘parent/adult together with child’), the actual frequency of brushing their child’s teeth (‘once or less a day’ or ‘twice or more a day’), the believed ideal frequency of brushing their child’s teeth and the frequency of forgetting to brush their child’s teeth (never, once to three times a week, four to seven times a week or more than seven times a week). Also, one item was included about the frequency of brushing the parent’s own teeth and the frequency of the parent visiting the oral health practitioner themselves (‘once or less a year’ or ‘twice or more a year’).

### Statistical analysis

The data were analysed using IBM SPSS Statistics for Macintosh, version 28 (IBM Corp., Armonk, N.Y. USA). Independent samples t-tests were used to compare the mean HAPA scores, child brushing frequency per day, mean dmft + DMFT, and mean age of the child and the mother at baseline between the IVS and BGT groups. Furthermore, we compared these variables between parents that completed all three questionnaires (T1, T2 and T3) and the parents that completed only the first questionnaire (T1) to reveal potential differences between these parents. Likewise, differences in categorical baseline variables between the IVS and BGT group and between parents that completed all three questionnaires (T1, T2 and T3) and the parents that completed the first questionnaire only (T1) were assessed using the chi-square test. For baseline comparisons significance levels were set at 5%.

The changes in mean HAPA scores during the treatment period were assessed with repeated measures ANOVA with post hoc LSD pairwise comparisons for each treatment group. The *within-subjects factor* was time (before, during and after treatment). The data were checked for violations of sphericity with Mauchly’s test of Sphericity. If the sphericity was violated (*p* < 0.05), the Greenhouse–Geisser correction was used. The significance level for ANOVA was set at 5%.

## Results

### Sample

**Fig. 3:**
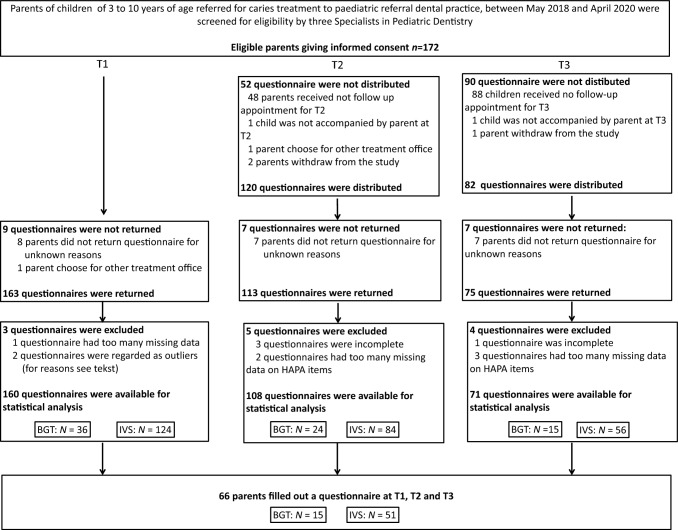
Flow chart data collection. *T2 at the check-up appointment after the treatment under intravenous sedation, or during the last treatment session with behavioural guidance techniques. **T3 at check-up appointment three months after dental rehabilitation

Data were collected from May 2018 to April 2020. Out of the 172 parents eligible for participation, 163 parents filled out questionnaires at T1. One questionnaire was removed because it had more than thirteen missing values on the HAPA items and we considered it as non-reliable. Two questionnaires were detected as outliers and removed from the data using outlier detection for ordered rating scales data (Zijlstra et al. 2013). The flowchart of the data collection (Fig. 3) illustrated that a total of 160 questionnaires were available for statistical analysis at T1, 108 questionnaires at T2 and 71 questionnaires at T3. Of these, 66 parents filled out a questionnaire at all three measurement times. There was a premature stop of the data collection during the course of the COVID-19 pandemic in 2020. The time between T1, T2 and T3 varied.

Descriptive characteristics and sociodemographic variables at baseline are shown in Tables 1 and 2. At baseline, the parents who filled out the questionnaire were mostly mothers 74.7%), 126 parents lived with a partner (82.4%), and 66% of the mothers were categorised as having a low level of education. The age of the mothers ranged from 22 to 48 years with a mean age of 34.2 years (SD = 5.6). Slightly more than half of the children (54.4%) were boys (*n* = 87), the children had a mean age of 5.2 years (SD = 1.5, range 3–9) and had a mean dmft + DMFT of 8.4 (SD = 2.4, range 3–14). More than half of the children had Dutch ethnicity (56.9%). Most of the children were treated under IVS (77.5%). Reasons for treatment under IVS are presented in Table 1. The children of the parents who filled out all three questionnaires (T1, T2 and T3) were older at baseline (5.5 years, SD = 1.6) compared to the children of the parents who filled out the questionnaire at baseline only (T1) (5.0 years, SD = 1.4, *p* = 0.046). The questionnaire was filled out by the same parent in 83.0% of the cases at T2 and in 75.8% at T3. The other descriptive characteristics and socio-demographic variables did not differ between parents who only filled out questionnaires at T1 and parents who filled out questionnaires at T1, T2 and T3.

**Table 1:** Baseline Characteristics of parents and their children of the total sample (*n* = 160) and comparisons between BGT (*n* = 36) and IVS (*n* = 124) (*X*^*2*^-test)

Covariate category	Total group *N* (%)*	BGT *N* (%)	IVS*N* (%)	*p *value
Relation to child Mother	118 (74.7)	27 (75.0)	91 (74.6)	0.314
Father	35 (22.2)	7 (19.4)	28 (23.0)	
Father & mother	4 (2.5)	1 (2.8)	3 (2.5)	
Other	1 (0.6)	1 (2.8)	0 (0)	
Parent’s/caregiver’s Marital status				
Two parents (married, living together)	126 (82.4)	31 (86.1)	95 (81.2)	0.499
One parent (single, divorced, widowed)	27 (17.6)	5 (13.9)	22 (18.8)	
Mother's educational level				
Low level of education	99 (66.0)	22 (62.9)	77(67.0)	0.654
High level of education	51(34.0)	13 (37.1)	38 (33.0)	
Child's gender				
Boy	87 (54.4)	22 (61.1)	65 (52.4)	0.357
Girl	73 (45.6)	14 (38.9)	59 (47.6)	
Child's ethnical background*				
Dutch	91 (56.9)	19 (52.8)	72 (58.1)	0.622
Non-Western	52 (32.5)	14 (38.9)	38 (30.6)	
Western	17 (10.6)	3 (8.3)	14 (11.3)	
Actual brushing frequency child a week				
Once or less a day	47 (29.6)	8 (22.9)	39 (31.5)	0.325
Twice or more a day	112 (70.4)	27 (77.1)	85 (68.5)	
Ideal brushing frequency child teeth’s				
Once or less a day	7 (4.4)	1 (2.9)	6 (4.9)	0.608
Twice or more a day	151 (95.6)	34 (97.1)	117 (95.1)	
Forgotten to brush child’s teeth frequency never	109 (68.6)	26 (72.2)	83 (67.5)	0.770
Not every day	49 (30.8)	10 (27.8)	39 (31.7)	
1/day	1 (0.6)	0(0)	1 (0.8)	
Person that brushes child’s teeth Supervised (parent, Adult, adult& child)	116 (84.7)	26 (83.9)	90 (84.9)	0.888
Non supervised (child only)	21 (15.3)	5 (16.1)	16 (15.1)	
Parent’s/caregiver’s frequency visiting OHP				
Once or less a year	26 (16.7)	9 (25.7)	17 (14.0)	0.103
T wice or more a year	130 (83.3)	26 (74.3)	104 (86.0)	
Parent’s/caregiver’s own brushing frequency				
Once or less a day	32 (20.9)	5 (14.7)	27 (22.7)	0.313
Twice or more a day	121 (79.1)	29 (85.3)	92 (77.3)	
Reason for treatment under IVS (*n* = 123)				
Young age			6 (4.9)	
Extended treatment plan			45 (36.6)	
LCB			6 (4.9)	
Young age and extended treatment plan			61 (49.6)	
Extended treatment plan and LCB			3 (2.4)	
Young age and extended treatment plan and LCB			2 (1.6)	
Missing			1 (0.8)	

*BGT* behavioural guidance techniques. *IVS* intravenous sedation. *LCB* limited coping behaviour

^*^N (%) depends on response depends on responses (valid percentage)

^*^Ethnical background child is labelled as migration background according to Statistics Netherlands (Statistic Netherlands, 2021)

Dutch background: Child and both parents were born in the Netherlands

Western migration background: Child or mother or father originating from a country in Europe (excluding Turkey), North America and Oceania, or from Indonesia or Japan

Non-Western migration background: Child or mother or father originating from a country in Africa, South America or Asia (excl. Indonesia and Japan) or from Turkey

In case both parents are not born in the Netherlands, the country of birth of the mother is dominant

**Table 2:** Baseline mean scores of the HAPA constructs, brushing frequency per day, age, dmft + DMFT for the total sample and comparisons between the two treatment groups (independent samples t-test)

Variable	Total group		BGT		IVS		
HAPA construct	*N*	Mean (SD)	*N*	Mean (SD)	*N*	Mean	*p*-value
Outcome expectancies	156	3.09 (0.58)	36	3.10 (0.53)	120	3.09 (0.60)	0.955
Risk perceptions	159	3.41 (0.53)	36	3.31 (0.51)	123	3.44 (0.54)	0.170
Action self-efficacy	159	3.42 (0.68)	36	3.59 (0.54)	123	3.37 (0.71)	0.081
Intention	151	3.34 (0.54)	35	3.32 (0.51)	116	3.34 (0.55)	0.851
Coping self-efficacy	159	3.47 (0.51)	36	3.59 (0.50)	123	3.44 (0.50)	0.107
Action planning	153	3.27 (0.52)	34	3.29 (0.54)	119	3.26 (0.52)	0.804
Coping planning	155	2.93 (0.71)	35	3.09 (0.75)	120	2.88 (0.70)	0.129
Action control	151	2.69 (0.60)	34	2.60 (0.73)	117	2.72 (0.56)	0.338
Age mother	157	34.15 (5.63)	36	35.00 (5.84)	121	33.90 (5.58)	0.305
Age child	158	5.21 (1.50)	34	5.82 (1.68)	124	5.04 (1.41)	**0.007**
dmft + DMFT	160	8.36 (2.40)	36	6.11 (2.15)	124	9.02 (2.05)	** < 0.001**

*BGT* behavioural guidance techniques. *IVS* intravenous sedation

Significance at *p* < 0.05 are in bold

### Baseline comparison between groups

Baseline mean HAPA scores and standard deviations for the total group and comparisons between treatment groups are shown in Table 2. Children in the IVS group were significantly younger than children in the BGT group (5.0 years (SD = 1.4) versus 5.8 years (SD = 1.7), *t*(156) = 2.76, *p* = 0.007) and dmft + DMFT was significantly higher in children in the IVS group than in children in the BGT group (9.0 (SD = 2.1) versus 6.1 (SD = 2.1), *p* < 0.001). There were no differences in gender and ethnic background between children of both groups at baseline. There were no differences between parents of both groups regarding the, marital status, level of education of the mother, brushing frequency of the child, reporting of the ideal brushing frequency, the frequency the parent forgot the brush their children’s teeth, supervised toothbrushing and parental dental visits and parental own brushing frequency (Tables 1 and 2). Furthermore, the mean scores on the HAPA constructs were high and varied from 2.69 (SD = 0.60) action control) to 3.47 (SD = 0.51) (coping self-efficacy). There were no differences in mean HAPA scores between the IVS group and the BGT group at baseline (Table 2).

### Changes in mean scores of the HAPA constructs over time and interaction effects

Repeated measures ANOVA per treatment group with post hoc LSD pairwise comparisons (Table 3) revealed a small main effect of time in the BGT group in *action self-efficacy* (F_(2,26)_ = 4.81, *p* = 0.016, *η*^2^ = 0.26), namely a decrease from T1 to T2 (*p* = 0.027) and from T1 to T3 (*p* = 0.021), and in *coping self-efficacy* F_(2,28)_ = 3.37, *p* = 0.049, *η*^2^ = 0.19) a decrease between T1-T2 (*p* = 0.015). In the IVS group, a small main effect of time was observed in *action planning* (F_(2,94)_ = 3.22, *p* = 0.046, *η*^2^ = 0.06), namely a decrease between T1 and T3 (*p* = 0.036) and in *action control* (F_(2,98)_ = 5.50,* p* = 0.005, *η*^2^ = 0.010), namely an increase between T1-T2 (*p* = 0.012) and between T1-T3 (*p* = 0.006).

**Table 3:** Means and standard deviations of the HAPA constructs and brushing frequency per day per measurement time and the results of the repeated measures ANOVA per treatment group

		T1	T2	T3			Pairwise comparison
	*N*	Mean (SD)	Mean (SD)	Mean (SD)	*p-*value	*η2*	
Outcome expectancies							
BGT	15	3.12 (0.57)	3.09 (0.41)	3.22 (0.64)	0.568		
IVS	48	3.08 (0.68)	3.24 (0.51)	3.30 (0.46)	0.081		
Risk perception							
BGT	15	3.36 (0.53)	3.45 (0.62)	3.31 (0.58)	0.603		
IVS	50	3.46 (0.59)	3.41 (0.52)	3.32 (0.58)	0.343		
Action self-efficacy							
BGT	15	3.64 (0.61)	3.36 (0.61)	3.13 (074)	**0.016**	0.26	T1-T2 (*p* = 0.027),T1-T3 (*p* = 0.021)
IVS	51	3.49 (0.66)	3.58 (0.52)	3.53 (0.55)	0.533		
Intention							
BGT	14	3.24 (0.48)	3.01 (0.25)	3.10 (0.51)	0.214		
IVS	48	3.41 (0.60)	3.34 (0.52)	3.30 (0.50)	0.486		
Coping self-efficacy							
BGT	15	3.63 (0.51)	3.23 (0.59)	3.37 (0.63)	**0.049**	0.19	T1-T2 (*p* = 0.015)
IVS	50	3.49 (0.50)	3.45 (0.52)	3.39 (0.54)	0.321		
Action planning							
BGT	14	3.17 (0.59)	3.23 (0.51)	2.94 (0.83)	0.426		
IVS	48	3.25 (0.54)	3.23 (0.55)	3.05 (0.49)	**0.046**	0.06	T1-T3 (*p* = 0.036)
Coping planning							
BGT	14	2.97 (0.78)	2.79 (0.68)	2.66 (0.66)	0.407		
IVS	48	2.77 (0.66)	2.91 (0.60)	2.82 (0.56)	0.369		
Action control							
BGT	14	2.76 (0.75)	2.80 (0.39)	2.80 (0.69)	0.966		
IVS	50	2.58 (0.56)	2.82 (0.59)	2.87 (0.48)	**0.005**	0.10	T1-T2 (*p* = 0.012), T1-T3 (*p* = 0.006)

*IVS* intravenous sedation. *BGT* behavioural guidance techniques

significance at *p* < 0.05 are in bold

^g^ = Greenhouse–Geisser correction

*η2* = eta2[g] = partial eta squared

## Discussion

In the present study, we measured the social cognitive constructs of the Health Action Process Approach in parents of children with high caries occurrence referred to a paediatric dental referral practice. We had two goals. Firstly, to compare the baseline HAPA scores of two groups, namely parents of children to be treated under intravenous sedation (IVS) and parents of children to be treated with behavioural guidance techniques (BGT). Secondly, within each group, we compared the change in HAPA scores over time: before, immediately and three months after treatment. Our results showed that at baseline, the mean scores of the HAPA constructs were similar in both treatment groups. Over time, *action self-efficacy* (the confidence parents have in their capacity of starting the new healthy behaviour) and *coping self-efficacy* (parents’ belief in overcoming the obstacles that arise during the maintenance of the new behaviour) showed a small decrease in the BGT group and *action planning* (a step-by-step planning to perform the desired behaviour) marginally decreased in the IVS group. Furthermore, *action control* (a continuous cycle of adapting new behaviour, perceiving set-backs and recovering from set-backs) slightly increased for parents of children treated under IVS. Although these changes in HAPA scores were statistically significant, the changes were so small that they can probably be considered to be clinically irrelevant. A relevant addition of the present study to the current literature is that our data suggest that there are minimal differences in the HAPA constructs of the motivational and the volitional phase between parents of children treated under IVS and with BGT at all time points.

It was remarkable that the parents in our study had rather high scores on the HAPA constructs of the motivational and the volitional phase (e.g., ranging from 2.63 to 3.47 on a 4-point Likert scale), and that these mean scores were comparable for parents in both treatment groups at baseline. This suggests that all parents in our sample were motivated to brush their children’s teeth. It is unclear why this was the case. It might be that referral to the paediatric dentist initiated the search for remedies for their child’s disease what motivated them to brush. Another explanation might be that parents were motivated to brush, but perceived barriers to brush properly. Finally, it might be that the referring dentist already started to educate better oral health behaviour, which increased parents' motivation for tooth brushing.

Although 70.4% of all parents reported an desirable brushing frequency of two times a day, both groups had high caries experience compared to the mean caries experience of Dutch children (Schuller et al. 2018). It might be that parents were motivated or became motivated after the referral of their child or even increased their motivation after accepting participating in the study and started brushing their child’s teeth, but experienced barriers during tooth brushing, resulting in ineffective plaque removal. Marshman et al. (2016) also found that parents, even though they were motivated to brush their children’s teeth twice a day, experienced difficulties and barriers in implementing the desired behaviour, such as the child’s cooperation and hectic daily routine. Another explanation might be that these parents over-reported the frequency of brushing their children’s teeth or changed their brushing behaviour recently. An open-ended question on brushing behaviour might not reliably reflect parent’s oral health performances.

Against our expectations, the type of treatment hardly influenced the HAPA scores of parents. Parental awareness of the oral health situation could possibly be increased due to frequent visits to the dentist during dental rehabilitation with BGT. During these visits, the dentist can build a positive relationship with the child and parent. It is known that an emphatic health care provider, one who is able to take a patient’s point of view and has the capacity to sympathise with the emotion of another person, is essential for the success of medical treatments (Squier 1990) and the reduction of dental anxiety (Jones and Huggins 2014) A patient who trusts his or her physician, is more likely to adhere to treatment regimens and may thus have better treatment outcomes (Jahng et al. 2005; Martin et al. 2005; Brand et al. 2013; Jones and Huggins 2014). For these reasons, the social cognition of parents regarding tooth brushing may increase, which can reflect an increase in the mean HAPA scores after dental rehabilitation with BGT. Nevertheless, in our study the mean scores of most HAPA constructs in the motivational phase and volitional phase of parents of children treated with BGT did not increase during the dental rehabilitation period of the child, apart from a small increase in coping self-efficacy between T2 and T3. On the contrary, a small decrease was observed in *action self-efficacy*, *coping self-efficacy*. It might be that high mean scores at baseline impede measuring an increase in mean scores on the HAPA constructs. Another explanation could be that insufficient education on brushing their children’s teeth was provided to parents during the rehabilitation treatments of their child, or that this education was not tailored according to HAPA.

For the IVS group, we had expected that parents of children treated under deep sedation need additional support in implementing positive oral health behaviour (Aljafari et al. 2015). A study showed that parents continued their irregular attendance patterns of post-operative dental visits (Olley et al. 2011). In contrast, some parents of children treated under deep sedation get alarmed by the drastic intervention, feel responsible to improve the oral health of their children in the future and get motivated for improving their children’s oral health (Amin and Harrison 2009). In our view, however, too few or no post-operational visits may prevent the development of a positive relationship with the dental professional and the encouragement that parents need to bridge the intention-behaviour gap. These parents would, therefore, present no changes in motivational and volitional constructs after the treatment of their children. However, in our study, for the IVS group, a significant, but minimal decrease was observed in *action planning* and a minimal increase was observed in *action control*. Studies showed that a recent experience of dental treatment under general anaesthesia of a child can temporarily increase preventive oral health behaviour (Amin and Harrison 2009) and increase the children’s own awareness of oral health (Amin and Harrison 2007). As awareness is one of the factors of action control, it might reflect in higher scores of action control. On the other hand, parents of children treated under general anaesthesia lack follow-up appointments to receive guidance on bridging the gap from intention to oral health behaviour which might lead to a decrease in action planning. The dental team could take advantage of this window of opportunity before it fades out, i.e., by making efforts that the follow-up appointments after dental rehabilitation are used to guide parents and children towards sustainable new behaviour.

Our results need to be interpreted from the perspective of the limitations of the study. First, we performed multiple testing, which could have increased the chance of the type I error. Therefore, a statistically significant change in HAPA scores might be a statistical chance. Moreover, we consider the small changes found in our study to be clinically irrelevant. Secondly, the small standard deviation of the HAPA mean score of the total group signalled uniformity between the participants. This implies that differences in HAPA scores between any arranged subgroups would be small. Additionally, differences between the treatment groups only appeared for dmft + DMFT and the age of the child. It might be that the overall high caries experience of the children in our study, compared to the average Dutch child (Schuller et al. 2018), overruled any expected differences between groups. Also, in the imbalanced sample the parents in the IVS group, which was three time as large as the BGT group, might have dominated the results. If the BGT group had consisted of the same number of parents as the IVS group, the sample would have included older children and children with lower caries experience, which might have resulted in a statistical difference between the group. Thirdly, we could not collect all data immediately and three months after the treatment of the children due to the beginning of the COVID-19 pandemic. During the course of the pandemic, the children’s treatments continued. Data collection, however, was not always possible due to the Dutch measures against the coronavirus (Ministry of Health, Welfare and Sport 2020). This resulted in a smaller sample than initially aimed to compare groups at each measurement point over time. In addition, our sample size was based on measuring a difference in mean HAPA scores of 0.4 between groups in an unbalanced allocation. We considered this difference to be a clinically relevant effect. In our study, only two out of the seven differences in HAPA scores between groups were ≥ 0.4 and could be considered as clinically relevant. Fourthly, the study design did not include an intervention based on HAPA or any other behavioural change model. It is likely that a HAPA-based intervention increases parental motivation to brush children’s teeth as well as brushing frequency. Finally, it might be that the self-reported measure of *oral health behaviour* is too subjective. Although previous research showed that brushing (Pakpour and Sniehotta 2012) and flossing (Schüz et al. 2007) behaviour could be measured reliably with one open-ended question, a more objective measure for oral health behaviours, such as an index for of plaque, gingival bleeding or recorded brushing duration could be used or added to a study as an attempt to get more trustworthily results.

Overall, our findings showed that parents of high caries risk children, either undergoing treatment under IVS or with BGT, seemed to be highly motivated to improve their children’s oral health and brush their children. When parents are motivated to improve brushing their children’s teeth, it is up to the dental team to take all opportunities to turn parental motivation into positive oral health behaviour. Next to adding knowledge on brushing frequency and practicing effective plaque removal, members of the dental team should take responsibility and play an active role in guiding parents in positive oral health behaviour. This could be done by discussing the difficulties and barriers for implementing tooth brushing and assisting parents in overcoming those barriers. The HAPA model can be used to aid parents as well as the dental team in bridging the gap from intention to positive oral health behaviour. This could be done, for example, by assisting parents in designing a plan, giving alternatives to overcome barriers, keeping track of daily brushing, sending reminders of the goals parent have set. Therefore, a HAPA-based intervention aimed to increase the social cognition of parents of high caries risk children might be a useful next step to improve the oral health behaviour for their children.

## Conclusion

Considering any limitations of the present prospective cohort study, assessing the social cognitive constructs of the Health Action Process Approach, it has been shown that**:**

- Parents of high caries risk children referred for treatment to a paediatric dental referral practice seem to be motivated to brush their children’s teeth, irrespective of the treatment being provided under intravenous sedation or with behavioural guidance techniques.

- The small changes in the Health Action Process Approach scores observed during the rehabilitation treatment period were not considered as clinically relevant.

### Supplementary Information

Below is the link to the electronic supplementary material.

Supplementary file1 (DOCX 27 KB)

## Data Availability

The data that support the findings of this study are openly available in Figshare, https://doi.org/10.6084/m9.figshare.20102627.
