# Supplementary material for: Are parents of high caries risk Dutch children motivated to brush their children’s teeth? An assessment using the health action process approach questionnaire
Source: Eur Arch Paediatr Dent. 2023 Jul 23;24(5):591–601. doi: 10.1007/s40368-023-00823-0 (PMC10600302; doi:10.1007/s40368-023-00823-0)
Supplement: Supplementary file 1 — Supplementary file1 (DOCX 27 KB) [file 40368_2023_823_MOESM1_ESM.docx]

Online supplementary information S1 Questionnaire with HAPA-items (In English)

**Identification number: ________**

Year of birth**:** ………………………….(yyyy)

**Socio-demographic characteristics**

***These questions are related to the abovementioned child***

1. What is the age of your child? …………..
2. What is the gender of your child?:

- *male^1^*
- *female^2^*

1. What is your relationship with the child? (tick one box only)

- *father^1^*
- *mother^2^*
- *other ^3^,…………………*

1. How many children do you have? (tick one box only)

- *1 child*
- *2 children^2^*
- *3 children^3^*
- *4 children^4^*
- *5 children^5^*
- *>5 children^6^*

1. How many older siblings does your child have? (tick one box only)

- *this is the oldest child^1^*
- *1 older sibling^2^*
- *2 older siblings ^3^*
- *3 older siblings ^4^*
- *4 older siblings ^5^*
- *5 older siblings ^6^*

1. With whom does your child live usually? (tick one box only)

- *with the father^1^*
- *with the mother^2^*
- *in one home with the father and mother in one home^3^*
- *alternately with the father and the mother ^4^*
- *with mother and stephfather^5^*
- *with father and stephmother^6^*
- *with grandparents^7^*
- *with other family member^8^, namely………………………………*
- *other,^9^ namely……………………*

1. What is the country of birth of your child? …………………………
2. Did your child ever need teeth pulled? (tick one box only)

- *no, never ^1^*
- *yes, ever or in the near future^2^*

1. Did your child ever need a teeth filled or replaced? (tick one box only)

- *no, never^1^*
- *yes, ever or in the near future^2^*

1. Did you child ever had dental treatment under deep sedation?

- *no, never^1^*
- *yes, date………^2^*

1. If yes, where did the treatment take place?...............
2. How would you describe the condition of your child’s natural teeth? (tick one box only)

- *excellent^1^*
- *very good^2^*
- *good^3^*
- *fair ^4^*
- *poor^5^*

**The following question are related to your ideas about oral health of your child. There are no true of false answers, it is about your opinion. Please, thick one box for each statement.**

| In the period ahead, I intend to…. | absolutely not true | not true | true | absolutely true |
| --- | --- | --- | --- | --- |
| 1. … to brush my child’s teeth properly once a day |  |  |  |  |
| 1. ... brush my child’s teeth myself |  |  |  |  |
| 1. … check my child's teeth after brushing |  |  |  |  |

| If I brush my child’s teeth on a daily basis… | most unlikely | unlikely | likely | most likely |
| --- | --- | --- | --- | --- |
| 1. …people in my community will see that my child is a clean person |  |  |  |  |
| 1. ...my child will remain having healthy teeth |  |  |  |  |
| 1. ...my child will feel good with beautiful teeth |  |  |  |  |

| If I don’t brush my child’s teeth daily then… |  |  |  |  |
| --- | --- | --- | --- | --- |
| 1. ...my child will be at risk for developing gum diseases |  |  |  |  |
| 1. ...my child will be at risk for developing tooth decay |  |  |  |  |
| 1. ...then the new permanent teeth will be harmed |  |  |  |  |
| 1. ...then my child might lose his/her teeth too soon |  |  |  |  |
| 1. ...then my child might have bad breath |  |  |  |  |

| I am confident that I immediately can start brushing my child’s teeth daily… | absolutely not true | not true | true | absolutely ture |
| --- | --- | --- | --- | --- |
| 1. ...even if I have to force myself to do so |  |  |  |  |
| 1. ...even if it is time consuming |  |  |  |  |
| 1. ...even if others do not brush their children’s teeth |  |  |  |  |

| I am confident that I can continue daily brushing my child’s teeth … |  |  |  |  |
| --- | --- | --- | --- | --- |
| 1. ...even when I cannot see any positive changes immediately |  |  |  |  |
| 1. ...even when my child does not cooperate |  |  |  |  |
| 1. ...even when I am in a hurry |  |  |  |  |
| 1. ...even when it takes a long time to become part of my routine |  |  |  |  |

| I have made a concrete plan… |  |  |  |  |
| --- | --- | --- | --- | --- |
| 1. ….where to brush my child’s teeth |  |  |  |  |
| 1. ….when to brush my child’s teeth |  |  |  |  |
| 1. ...how often to brush my child’s teeth |  |  |  |  |
| 1. ...how to brush my child’s teeth |  |  |  |  |
| 1. ...how much time to spend with brushing my child’s teeth |  |  |  |  |

| To keep brushing my child's teeth in difficult situations, I have made a concrete plan… |  |  |  |  |
| --- | --- | --- | --- | --- |
| 1. ...in case something interferes with brushing my child’s teeth |  |  |  |  |
| 1. ...in case I am in a hurry |  |  |  |  |
| 1. ...in case my child does not cooperate |  |  |  |  |
| 1. …in case my child has pain. bleedings gums or tooth decay |  |  |  |  |

| In the past week… |  |  |  |  |
| --- | --- | --- | --- | --- |
| 1. …I have consistently monitored how. when and how often I have brushed my child’s teeth |  |  |  |  |
| 1. … I have kept track of what prevented me from brushing my child’s teeth’, |  |  |  |  |
| 1. … **my child** carefully kept track of how often I brushed his/her teeth |  |  |  |  |
| 1. ...I have been very involved in brushing my child’s teeth |  |  |  |  |
| 1. ... I really tried to reach the goals that I have set for brushing my child’s teeth’ |  |  |  |  |

**The general advise is to brush teeth daily. This might be forgotten. Can you indicate:**

1. How many times a day did you brush your own teeth in the past week? (tick one box only)

- *never^1^*
- *less than once a day^2^*
- *once a day^3^*
- *twice a day^4^*
- *more than twice a day^5^*

1. How many times a day do you want your child’s teeth to be brushed? (tick one box only)

- *never^1^*
- *less than once a day^2^*
- *once a day^3^*
- *twice a day^4^*
- *more than twice a day^5^*

1. How many times in the past week your child’s teeth were brushed at least?(tick one box only)

- *never^1^*
- *less than once a day^2^*
- *once a day^3^*
- *twice a day^4^*
- *more than twice a day^5^*

1. How many times in the past week your child’s teeth were forgotten to be brushed? (tick one box only)

- *never^1^*
- *1-3 times^2^*
- *4-7 times^3^*
- *> 7 times ^4^*

1. Who brushes your child’s teeth? (tick one box only)

- *a parent^1^*
- *my child^2^*
- *first my child, then a parent^3^*
- *first a parent, then my child^4^*
- *other^5^,namely……………………*

1. How often do you visit the dentist or oral hygienist? (tick one box only)

- *never^1^*
- *only for complaints^2^*
- *once every two years^3^*
- *once every year^4^*
- *twice a year^5^*
- *more than twice a year^6^*

***The next questions are related to you***

|  |  |  |  |
| --- | --- | --- | --- |

1. What are the four number of your postal code:
2. What is the age of the mother of the child? ………………………
3. What is your ethnicity? (tick one box only)

- *Netherlands^1^*
- *European Union, other than Netherlands^2^*
- *Surinamese-Hindustani^3^*
- *Surinamese-Creole^4^*
- *Cape Verdean^5^*
- *Dutch Antillian^6^*
- *Moroccan^7^*
- *Turkish^8^*
- *American^9^*
- *African^10^*
- *Asian^11^*
- *other^12^, namely ………………………………………….*
- *not applicable/ I do not wish to answer^13^*

1. Which culture you feel comfortable?...................................................
2. What is the country of birth of the mother of the child:…………………….
3. What is the country of birth of the father of the child:………………………..
4. What is the highest education level completed by the mother of the child? (tick one box only)

- *University^7^*
- *Further education at a higher level (in the Netherlands ‘HBO’)^6^*
- *Secondary school at a higher level (in the Netherlands ‘HAVO’ or ‘VWO’)^5^*
- *Further education at a lower level (in the Netherlands ‘LBO’ or ‘MBO’)^4^*
- *Secondary school at a lower level (in the Netherlands ‘VBO’, ‘VMBO’ or ‘MAVO’)^3^*
- *Elementary school^2^*
- *No education^1^*
- *I don’t know^8^*
- *Not applicable/ I do not wish to answer^9^*

1. What kind of employment do you have? (tick one box only)

- *full time(35 hours or more) ^1^*
- *part time ( 12-35 hours) ^2^*
- *part time (0-12 hours)^3^*
- *no work^4^*
- *health insurance act ^5^*
- *not applicable/ I do not wish to answer^6^*

1. What is your relationship status ? (tick one box only)

- *Married^1^*
- *Living together with partner^2^*
- *Single/never been married^3^*
- *Divorced/separated^4^*
- *Widowed^5^*
- *Not applicable/ I do not wish to answer^6^*

**Please, take some time to check if you have completed all questions and signed the consent form.**

**Thank you for your cooperation!**
